# Bromodomain protein 4 is a novel predictor of survival for gastric carcinoma

**DOI:** 10.18632/oncotarget.16087

**Published:** 2017-03-10

**Authors:** Yixin Zhu, Weijin Yang, Guangnian Ji, Nan Lin, Weihang Wu, Ping Xiong, Chenxin Zheng, Lei Yan, Peng Wan, Yu Wang

**Affiliations:** ^1^ Clinical Institute of Fuzhou General Hospital, Fujian Medical University, Fuzhou, Fujian 350025, China; ^2^ Department of General Surgery, Dongfang Hospital, Xiamen University, Fuzhou, Fujian 350025, China; ^3^ Department of General Surgery, Fuzhou General Hospital, Fuzhou 350025, China; ^4^ Dongfang Hospital Affiliated to Xiamen University, Xiamen University, Xiamen, Fujian 361005, China

**Keywords:** BRD4, gastric adenocarcinoma, surgical resection, predictor, prognosis

## Abstract

Expression of bromodomain protein 4 (BRD4) has been reported to predict a worse prognosis in solid tumors. However, its expression profile and prognostic value in gastric carcinoma (GC) remains unknown. Here we investigated BRD4 expression in GC and explored its association with patient survival. Tissue samples were obtained from 95 GC patients who underwent surgical resection to remove the primary tumor from January 2009 to December 2010. Immunohistochemistry was used to detect the expression of BRD4 in GC tissues and adjacent normal tissues. Kaplan–Meier survival curves and Cox proportional hazards regression were used to analyze the data of BRD4 expression profile and clinicopathological characteristics. Immunohistochemical analysis revealed BRD4 was overexpressed in GC tissue compared with adjacent normal tissue. BRD4 expression was significantly associated with TNM stage (*p* < 0.001), lymphatic permeation (*p* = 0.011), and vital status at the end of follow-up (*p* < 0.001). Kaplan–Meier survival curves and the log-rank test demonstrated that higher BRD4 expression was an adverse predictive factor for survival in GC. Multivariate analysis by Cox proportional hazards regression revealed that BRD4 expression was an independent worse prognostic factor in GC. In conclusion, BRD4 could act as a potential biomarker for prognostic assessment of GC.

## INTRODUCTION

Gastric carcinoma (GC) is one of the most common malignant tumors of the digestive system. GC is the third most frequent cause of cancer-related death worldwide and affects approximately one million people every year [1]. On a global scale, the incidence ratio of GC between males and females is ~2:1. The incidence of GC in Asia is markedly higher than in Europe, and Northeast Asia has the highest incidence of GC worldwide—up to 69 cases per 100 000 people per year [2]. In particular, a large number of new GC cases (approximately half of the world's total number of GC cases) are diagnosed each year in China [3]. Chinese patients with advanced GC have a low 5-year survival rate of only ~25% [4].

Existing treatments for GC, including invasive techniques such as laparoscopic resections combined with chemotherapy or radiotherapy, have proven to be effective in prolonging the overall survival time mainly in patients at an early stage [5]. For advanced GC, it is difficult to achieve locoregional control with merely laparoscopic resections. While there are many reasons for the overall poor prognosis in GC, low rates of early diagnosis is a major contributing factor. Additionally, available chemotherapy treatments have a poor effect on patients with advanced GC [6]. Targeted therapies for GC such as HER2 inhibitors (e.g., trastuzumab) and a VEGF inhibitor (ramucirumab) are also now clinically accepted and have shown positive results in clinical trials [7]. However, a considerable portion of patients with advanced GC fail to benefit from these targeted therapies since tumors can develop resistance [8]. Therefore, there is an urgent need to identify a novel therapeutic target in advanced GC to provide better treatment options.

The bromodomain (BRD) and extra terminal domain (BET) family is composed of three ubiquitously expressed proteins, BRD4, BRD3, and BRD2, and a testis-specific protein, BRDT. The BET protein family members bind to acetylated lysine residues in histones and function as epigenomic readers at the interface between chromatin remodeling and transcription regulation [9, 10]. Particularly, BRD4 can regulate cell growth that leads to the development and progression of many diseases including cancer [11]. For example, BRD4 was shown to promote the growth and proliferation of hepatocarcinoma cells, enhance their ability to invade distant organs, and facilitate epithelial-mesenchymal transition in hepatocellular carcinoma [12]. BRD4 also functions in the inflammatory response by enhancing transcriptional activation of NF-kB and the expression of a subset of NF-kB-dependent inflammatory genes [13]. In *in vitro* studies, two BRD4 inhibitors (JQ1 and I-BET762) have been found to effectively inhibit the proliferation and growth of cancer cells such as melanoma, pancreatic cancer, lung cancer, multiple myeloma, acute myeloid leukemia, and Burkitt's lymphoma [14, 19, 21–23]. Although BRD4 has been demonstrated to act as a potential therapeutic target for several cancers, it has not yet been investigated in GC.

In the present study, we hypothesized that high expression of human BRD4 protein may promote the proliferation and invasion of GC cells. To test this hypothesis, we investigated BRD4 expression in GC by immunohistochemistry and explored its association with prognosis in patients with GC.

## RESULTS

### Clinicopathological characteristics of GC patients

This study included 95 patients who had undergone surgical resection of GC (and whose diagnosis was confirmed as GC by more than two pathologists). In total, 41.1% of included patients were aged less than 60 years and 58.9% were 60 years or older. Among these, 73.7% were men and 26.3% were women. Approximately half (51.6%) of the patients had a smoking or drinking history. Detailed clinicopathological characteristics of the included GC patients are listed in Table 1.

**Table 1:** Clinicopathological characteristics of patients with gastric carcinoma

Clinicopathological characteristics	Number of cases (*n* = 95)	
	Number of cases	%
**Age (years)**		
< 60	39	41.1
≥ 60	56	58.9
**Gender**		
Male	70	73.7
Female	25	26.3
**Smoking or drinking**		
No	46	48.4
Yes	49	51.6
**Histologic grade**		
Well differentiated	8	8.4
Moderately differentiated	47	49.5
Poorly differentiated	40	42.1
**TNM stage**		
T1	20	21.6
T2	20	21.6
T3	21	22.1
T4	34	35.7
**Lymphatic permeation**		
Absent	34	35.8
Present	61	64.2
**Tumor shape**		
Ulcerative type	73	76.8
Papillary type	1	1.1
Superficial type	11	11.6
Protrude type	5	5.3
Massive type	2	2.1
Infiltrative type	3	3.2
**Histologic type**		
Adenocarcinoma	86	90.5
Signet-ring cell carcinoma	9	9.5
**Distant metastasis**		
No	68	71.6
Yes	27	28.4
**EGFR expression**		
Low	73	78.5
High	20	21.5
**BRD4 expression**		
Low	50	52.6
High	45	47.4
**Vital status (at the end of follow-up)**		
Alive	39	41.1
Dead	56	58.9

In terms of histologic grade, a few cases were well differentiated (8.4%), while most cases were moderately (49.5%) or poorly differentiated (42.1%). For the T classification, T1, T2, and T3 accounted for similar proportions (~22% each); the majority of cases were T4 (35.7%). Lymphatic permeation was present in 64.2% of the patients. Among the six different tumor shapes, the ulcerative type was most common (76.8%). The vast majority (90.5%) were classified as adenocarcinoma, while the remaining cases were signet-ring cell carcinoma. Approximately 28.4% of the patients had distant metastasis. High EGFR and BRD4 expression was recorded in 21.5% and 47.4% of the patients, respectively. After surgery, 41.1% of the patients were alive at the end of the follow-up.

### BRD4 expression and its relationship with clinicopathological characteristics in GC patients

**Figure 1:**
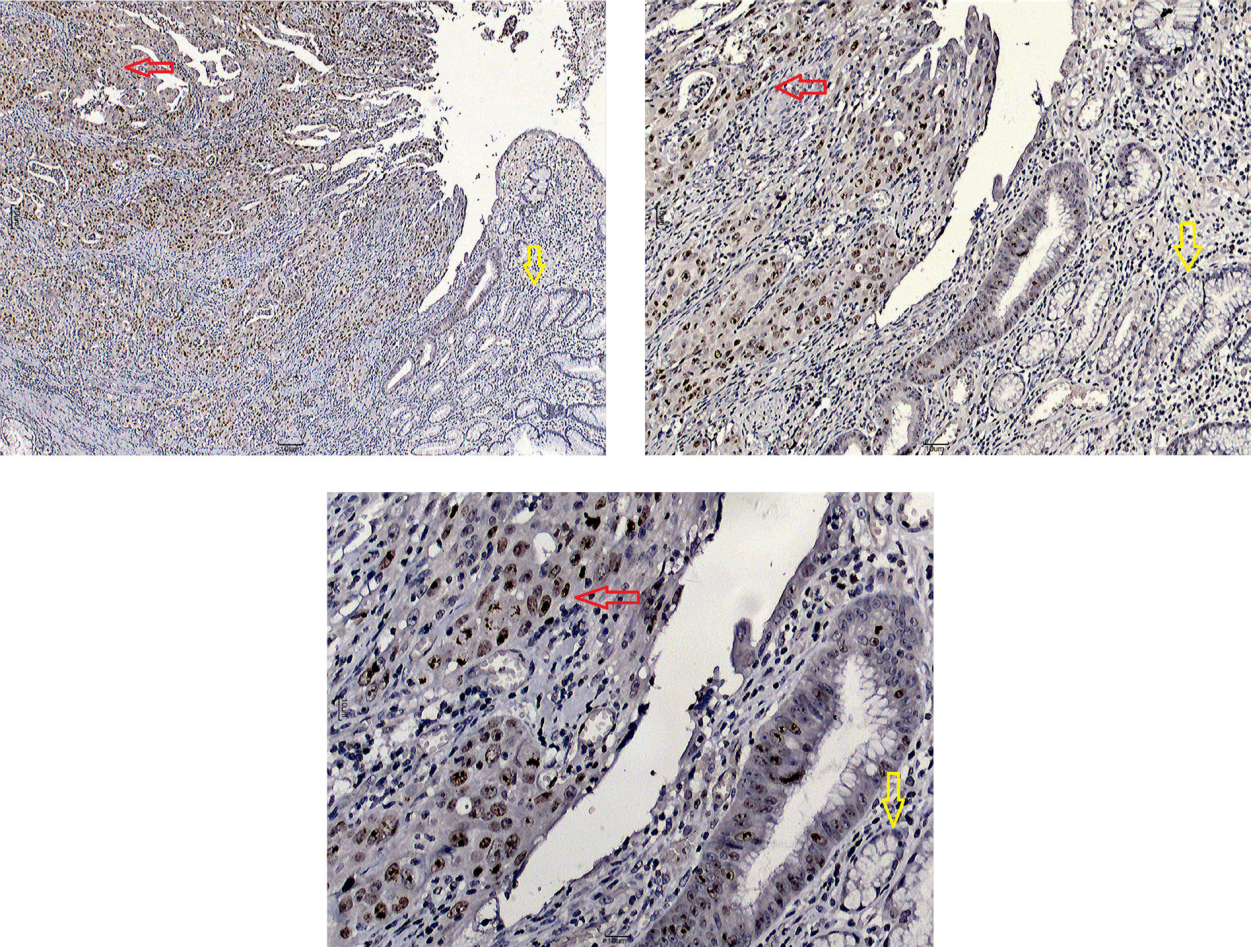
Immunohistochemical staining of BRD4 protein in gastric carcinoma (red arrow) and adjacent normal tissue (yellow arrow) Tissue samples were observed under an Olympus CX41 light microscope (Tokyo, Japan). Digital images were obtained using Moticam 2206 (Motic Instruments, Richmond, British Columbia, Canada) and Motic Images Advanced 3.2 software at different magnifications.

To detect the expression level of BRD4 in human GC, tissue samples were selected from 95 GC cases (confirmed following surgery) and examined by immunohistochemistry. We determined the immunoreactive score by the sum of BRD4 expression extension and intensity [26]. Immunohistochemical staining indicated that BRD4 protein mainly accumulated in the nuclei of GC cells. The expression of BRD4 in GC tissues was markedly higher than in the adjacent normal tissue (Figure 1).

The relationship between clinicopathological characteristics and BRD4 expression in the GC patients is shown in Table 2. BRD4 expression was significantly associated with TNM stage (*p <* 0.001), vital status at the end of follow-up (*p <* 0.001), and lymphatic permeation (*p <* 0.05). No significant difference in BRD4 expression was observed with respect to gender, age, drinking or smoking status, histologic type, or EGFR expression (*p* > 0.05).

**Table 2:** Relationship between clinicopathological characteristics and BRD4 expression in patients with gastric carcinoma

Characteristics	BRD4 expression		*p* value
	Low (%)	High (%)	
**Age (years)**			
< 60	21 (53.8)	18 (46.2)	1.000
≥ 60	29 (51.8)	27 (48.2)	
**Gender**			
Male	38 (54.3)	32 (45.7)	0.645
Female	12 (48.0)	13 (52.0)	
**Drinking or smoking**			
No	27 (58.7)	19 (41.3)	0.306
Yes	23 (46.9)	26 (53.1)	
**TNM stage**			
T1	17 (85.0)	3 (15.0)	<0.001
T2	15 (75.0)	5 (25.0)	
T3	10 (47.6)	11 (52.4)	
T4	8 (23.5)	26 (76.5)	
**Lymphatic permeation**			
Absent	24 (70.6)	10 (29.4)	0.011
Present	26 (42.6)	35 (57.4)	
**Histologic type**			
Adenocarcinoma	46 (53.5)	40 (46.5)	0.731
Signet-ring cell carcinoma	4 (44.4)	5 (55.6)	
**EGFR expression**			
Low	37 (50.7)	36 (49.3)	0.804
High	11 (55.0)	9 (45.0)	
**Vital status (at the end of follow-up)**			
Alive	35 (89.7)	4 (10.3)	< 0.001
Dead	15 (26.8)	41 (73.2)	

Note: Bold values are significant at *p <* 0.05.

### Correlation between clinicopathological characteristics, BRD4 expression, and survival in GC patients

**Figure 2:**
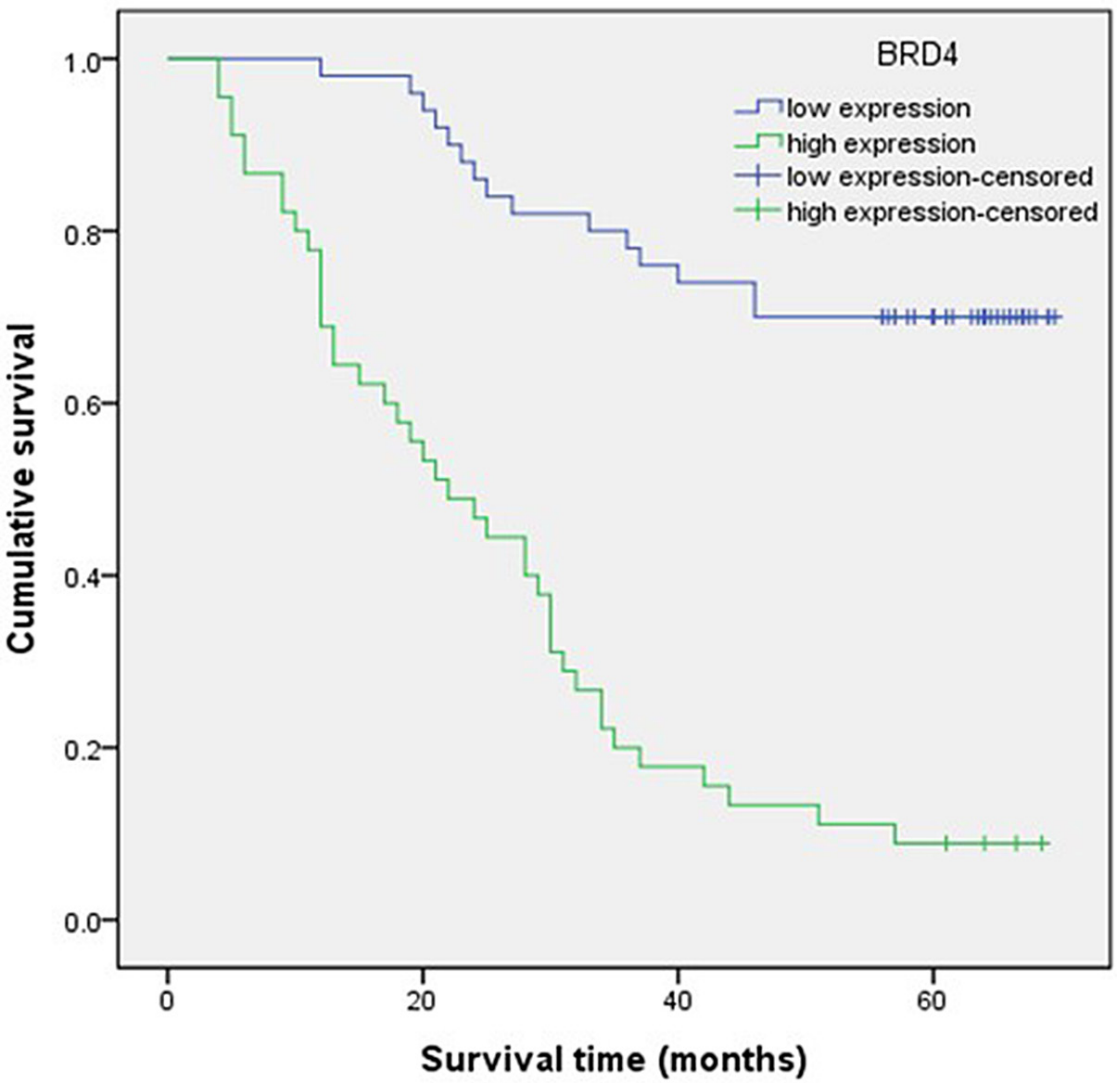
Survival analysis of patients with gastric carcinoma by the Kaplan–Meier method Patients with higher BRD4 expression in tumor tissue were closely correlated with poorer overall survival than patients with lower expression (*p <* 0.05).

Cumulative survival curves were calculated in the univariate survival analyses according to the Kaplan–Meier method plus log rank test. Univariate analysis demonstrated that BRD4 expression (*p <* 0.001), lymph node metastasis (*p <* 0.001), lymphatic permeation (*p <* 0.001), and TNM stage (*p <* 0.001) were significant prognostic factors for poor survival (Table 3). Kaplan–Meier analysis revealed that higher expression of BRD4 was correlated with adverse survival (Figure 2), and might act as an adverse prognostic predictor in GC patient. Multivariate analysis indicated that high BRD4 expression (*p <* 0.001) and advanced TNM stage (T2–T4, *p* = 0.007) were independent worse prognostic factors for overall survival in GC patients (Table 3). These results further demonstrate that high BRD4 expression was associated with adverse prognosis in GC patients.

**Table 3:** Univariate and multivariate Cox regression analysis of overall survival in patients with gastric carcinoma

Characteristics	Univariate analysis		Multivariate analysis	
	HR (95% CI)	*p* value	HR (95% CI)	*p* value
**Age (years)**				
< 60	1	0.185		
≥ 60	0.690 (0.399–1.194)			
**Gender**				
Male	1	0.104		
Female	1.593 (0.909–2.794)			
**Smoking or drinking**				
No	1	0.376		
Yes	1.270 (0.749–2.153)			
**pT stage**				
pT1	1	< 0.001	1	0.007
pT2	1.495 (0.474–4.711)		2.616 (0.566–12.100)	
pT3	4.811 (1.758–13.169)		4.609 (1.159–18.336)	
pT4	7.836 (2.997–20.489)		7.313 (1.944–27.517)	
**Histologic type**				
Adenocarcinoma	1	0.861		
Signet-ring cell carcinoma	1.085 (0.433–2.721)			
**Lymphatic permeation**				
Present	1	< 0.001	1	0.642
Absent	0.156 (0.070–0.346)		0.537 (0.039–7.387)	
**Lymph node metastasis**				
pN0	1	<0.001	1	0.507
pN1–pN3	6.932 (3.120–15.401)		2.455 (0.173–34.854)	
**EGFR expression**				
Low	1	0.361		
High	0.727 (0.367–1.442)			
**BRD4 expression**				
Low	1	< 0.001	1	< 0.001
High	6.387 (3.480–11.720)		3.859 (1.988–7.489)	

Note: Bold values are significant at *p <* 0.05.

## DISCUSSION

GC is a common malignant tumor of the digestive system and a frequent cause of cancer-related death worldwide [1]. Total gastrectomy or subtotal gastrectomy plus lymph node dissection is generally considered to be effective for the treatment of early GC, whereas multidisciplinary therapeutic approaches are needed for advanced GC [27]. Although BRD4 has been reported as a potential therapeutic target for several cancers, its role in GC is still unknown.

High expression of BRD4 has been reported in colorectal cancer, melanoma, metastatic breast cancer, hepatocellular carcinoma, and non-small cell lung cancer [17–20]. In the present study, we obtained the first evidence that BRD4 is also overexpressed in GC tissues compared with adjacent normal tissues. We have selected the primary monoclonal rabbit anti-human BRD4 antibody (1:200; Abcam, Cambridge, UK) according to a recent study on urothelial carcinoma of the bladder [25]. The study well demonstrated that the antibody was available for detecting the expression of BRD4. According to the above study, BRD4 protein was mainly accumulated in the nuclei of GC cells. If immunohistochemical staining found nothing in the nuclei of cells, it was thought to be false-positive and excluded. We tried different concentrations of antibody, ranging from 1:50 to 1:500. Finally, we found that 1:200 was the most suitable antibody concentration for the detection of BRD4, which was consistent with the results of other studies. The accumulation of BRD4 protein mainly observed in the nuclei of GC cells may contribute to cell proliferation. BRD4 has been hypothesized to be involved in cell cycle progression, as its binding to acetylated chromatin persists even during mitosis when chromatin is highly condensed and transcription is interrupted [18]. Additionally, we found that high expression of BRD4 was significantly associated with advanced TNM stage and adverse prognosis of GC. This indicates that high BRD4 expression may promote GC cell invasion and act as an adverse prognostic predictor of survival in GC patients. However, we found no association between the expression of BRD4 and EGFR, even though EGFR can promote GC cell invasion, and EGFR inhibitors can suppress EGFR activation in tumors [31]. Further *in vivo* study is needed to verify the relationship between BRD4, EGFR, and survival in GC.

Suppression of BRD4 expression using short hairpin RNAs or small-molecule inhibitors has shown anti-cancer effects [29, 30]. JQ1 is one of the most thoroughly studied BRD4 inhibitors. For example, *in vitro* studies have shown JQ1 has a modest effect on the clonogenic capacity of mobilized haematopoietic stem cells and almost completely inhibits leukemia-initiating cells. Furthermore, treatment with JQ1 at the doses required for significant anti-tumor activity does not lead to widespread systemic toxicity [10]. Additionally, JQ1 has been shown to be a potentially effective chemotherapeutic agent against human thyroid cancer [30]. In future, we will investigate JQ1 as a BRD4 inhibitor in GC, and then use this compound to determine whether BRD4 is involved in GC cell growth and invasion *in vitro*.

In summary, we report the first evidence that BRD4 is overexpressed in clinical GC tissues compared with adjacent normal tissues. Evaluation of BRD4 expression has clinical value for predicting the prognosis of GC, and high expression of BRD4 may indicate poor survival in GC patients. The results suggest that BRD4 overexpression is useful as a novel prognostic factor for GC patients.

## MATERIALS AND METHODS

### Patients, follow-up, and clinical characteristics

Ninety-five pathologically confirmed GC patients who had received curative resection in the General Surgery Institute, Fuzhou General Hospital (Fuzhou, China) between January 2009 and December 2010 were included in this study. TNM stage and histology were classified according to the 2002 AJCC staging system [24]. Exclusion criteria were patients with other serious diseases, such as heart or liver problems, those who had received preoperative chemotherapy or radiotherapy, and those without enough clinical information collected. Detailed medical records for all patients were collected from the hospital database. We define the survival time as the date from operation to the date of death or the end of follow-up. The study was approved by the Ethics Committee of Fuzhou General Hospital and all patients provided informed consent. All patients were followed up until December 2015.

### Immunohistochemistry

Tissue samples were collected from resected GC patients, fixed in 10% neutral formalin, and then embedded in paraffin. Paraffin sections (4 μm thick) were deparaffinized in xylene and rehydrated in grade alcohol, followed by boiling in 10 mmol/L of citrate buffer (pH 6.0) for antigen retrieval. The sections were blocked with 2% bovine serum albumin for 30 min and incubated overnight at 4°C with primary monoclonal rabbit anti-human BRD4 antibody (1:200; Abcam, Cambridge, UK) after the inhibition of endogenous peroxidase activities for 30 min with methanol containing 0.3% H_2_O_2_ [25]. After being washed three times with PBS, the slides were incubated with horseradish peroxidase-conjugated goat anti-rabbit IgG for 30 min according to the instruction of the UltraSensitiveTM S-P (Maixin, Fuzhou, China). Two pathologists, who were blinded to the clinicopathological information of the patients, independently evaluated the immunohistochemical staining. Immunoreactive scores were determined by the sum of BRD4 expression extension and intensity [26], a method commonly used by pathologists in our hospital and worldwide. According to the description by Yang et al. [25], the intensity of immunohistochemical staining was scored as: 0, no staining of the tumor cells; +, mild staining; ++, moderate staining; and +++, marked staining. The extension of immunohistochemical staining was evaluated and recorded as the percentage of the area of staining as following: 0, less than 5%; +, 5%–25%; ++, 26%–50%; +++, 51%–75%; and ++++, >75%. Thereafter, we combined the scores of staining intensity and extension as: -, 0; +, 1–2; ++, 3–5; and +++, 6–7. Cases scored as – or + were deemed as the low BRD4 expression group; and ++ or +++ were the high BRD4 expression group.

### Statistical analysis

The chi-square test was used to examine statistical associations between clinicopathological characteristics and BRD4 expression in GC patients. The relationship between BRD4 expression and overall survival was evaluated by the Kaplan–Meier method, and the differences between subgroups were evaluated by the log-rank test. Univariate and multivariate Cox proportional hazards regression model analyses were used to evaluate the prognostic value of known categorical variables and BRD4 expression. All statistical analyses were two-sided, and *p* values < 0.05 were regarded statistically significant. Computerized statistical analyses were performed using the Statistical Package for the Social Sciences (SPSS), version 18.0 (SPSS Inc., Chicago, IL, USA).
